# Low-Density Lipoprotein Cholesterol Is Independently Associated with White Matter Injury Beyond Coronary Artery Calcium: Insights into Brain Aging

**DOI:** 10.3390/jcm15093277

**Published:** 2026-04-25

**Authors:** Özgür Çakır, Burak Açar, Mustafa Kemal Dönmez, Almotasem Shatat, Sena Destan Bünül, Rıdvan Erten, Ahmet Yalnız, Ercüment Çiftçi

**Affiliations:** 1 Department of Radiology, Faculty of Medicine, Kocaeli University, Kocaeli 41380, Türkiye; mkemal.donmez@kocaeli.edu.tr (M.K.D.); almotasemsh@gmail.com (A.S.); ahmet.yalniz@kocaeli.edu.tr (A.Y.); ercument.ciftci@yahoo.com (E.Ç.); 2 Department of Cardiology, Faculty of Medicine, Kocaeli University, Kocaeli 41380, Türkiye; burakacarmd@yahoo.com; 3 Department of Neurology, Faculty of Medicine, Kocaeli University, Kocaeli 41380, Türkiye; destansena@gmail.com; 4 Division of Geriatrics, Department of Internal Medicine, Faculty of Medicine, Kocaeli University, Kocaeli 41380, Türkiye; ridvan.erten@kocaeli.edu.tr

**Keywords:** coronary artery calcium, LDL cholesterol, white matter hyperintensities, brain aging, heart–brain axis

## Abstract

**Background/Objectives**: The interplay between cardiovascular risk factors and brain aging remains incompletely understood. We aimed to investigate the comparative associations of coronary artery calcium (CAC) and low-density lipoprotein cholesterol (LDL-C) with MRI-derived volumetric measures of the brain. **Methods**: In this retrospective, single-center, cross-sectional study, 84 participants who underwent coronary computed tomography for CAC scoring and brain magnetic resonance imaging within 90 days were included; LDL-C levels were available in 69 participants for LDL-based analyses. Brain volumetric measures were obtained using the automated lesionBrain pipeline within the volBrain platform, which performs fully automated tissue segmentation and lesion quantification based on multi-atlas and patch-based approaches. Associations were evaluated using Spearman’s correlation with false discovery rate correction and hierarchical multivariable regression, supported by bootstrap validation and post hoc power analysis. The cohort had a mean age of 58.0 ± 13.0 years (range 19–78) and was derived from routine clinical imaging. **Results**: LDL-C was positively associated with abnormal white matter volume (ρ = 0.334, *p* = 0.005), although this did not remain statistically significant after FDR correction (pFDR = 0.090). In fully adjusted models, LDL-C remained the only independent predictor (β = 0.006, 95% CI: 0.002–0.010, *p* = 0.007; standardized β = 0.225; partial R^2^ = 11.7%), corresponding to a 6.2% increase in abnormal white matter volume per 10 mg/dL increase (derived from log-transformed models). CAC showed only a marginal association (*p* = 0.059). Post hoc power analysis demonstrated adequate power for LDL-C but insufficient power for CAC. Neither marker was associated with gray matter volume. **Conclusions**: In this cross-sectional cohort, higher LDL-C was independently associated with greater abnormal white matter volume after adjustment for cardiovascular risk factors, statin use, and CAC. No CAC–brain association was detected in this cohort, but limited statistical power means that small CAC effects cannot be excluded. These findings should be interpreted as associative rather than causal or mechanistic.

## 1. Introduction

Aging is accompanied by parallel structural and functional changes in both the cardiovascular and central nervous systems. Cardiovascular diseases and neurological disorders represent leading causes of morbidity and mortality, sharing common risk factors and overlapping pathophysiological mechanisms [1]. In this context, the concept of the heart–brain axis has gained increasing attention, emphasizing the close interrelationship between systemic vascular health and brain aging [2].

Coronary artery calcium (CAC) scoring, quantified using the Agatston method, is a well-established, non-invasive imaging marker of cumulative atherosclerotic burden [3]. CAC scoring is widely recommended for cardiovascular risk stratification and has consistently been shown to predict myocardial infarction, stroke, and cardiovascular mortality beyond traditional risk factors. Unlike transient metabolic markers, CAC reflects long-term exposure to vascular injury and established plaque calcification. However, whether this calcified burden directly contributes to cerebral structural damage or merely reflects prior atherosclerotic exposure remains uncertain [4,5].

Concurrently, structural brain changes represent a hallmark of aging. Even among cognitively normal older adults, progressive reductions in gray matter volume and the accumulation of white matter hyperintensities (WMHs) are commonly observed. These alterations are closely associated with cognitive decline and functional impairment. WMHs are widely regarded as a key radiological marker of cerebral small vessel disease, reflecting subtle microvascular injury that may precede overt neurodegeneration [6,7,8]. Accumulating evidence indicates that traditional cardiovascular risk factors, including hypertension, dyslipidemia, and elevated low-density cholesterol (LDL-C), contribute to adverse brain structural changes [9]. While CAC reflects the historical burden of calcified atherosclerosis, circulating lipids such as LDL-C may capture contemporaneous metabolic exposure that could be relevant to cerebral microvascular health [10,11].

Several studies have reported associations between higher CAC scores and cognitive decline [12,13]. However, investigations directly examining the relationship between CAC burden and quantitative brain volumetric measures have yielded inconsistent findings [14]. Prior neuroimaging studies have linked vascular calcification and atherosclerotic burden with white matter hyperintensities, covert infarction, and brain atrophy, although findings remain heterogeneous. Furthermore, the relative contributions of calcified plaque burden (CAC) versus active lipid markers (LDL-C) to structural brain aging remain understudied. To our knowledge, no study has directly compared CAC and LDL-C in relation to quantitative MRI-derived brain volumetric measures within the same cohort.

Accordingly, the present study aimed to investigate the associations of coronary artery calcium burden and serum LDL-C with MRI-derived brain volumetric measures in an adult population. Based on the conceptual distinction between cumulative calcified burden and contemporaneous lipid exposure, we hypothesized that LDL-C would show stronger associations with abnormal white matter volume than CAC.

We further examined associations across all brain compartments, including gray matter, to characterize the regional specificity of these relationships.

## 2. Materials and Methods

### 2.1. Study Design and Population

This retrospective, single-center, cross-sectional study was conducted at a tertiary-care university hospital between January 2020 and December 2023. These temporal windows were selected to balance clinical feasibility with temporal proximity, as structural brain measures are relatively stable over short intervals and LDL-C was intended to reflect near-contemporaneous metabolic status. Adult participants who underwent both non-contrast coronary computed tomography (CT) for coronary artery calcium (CAC) scoring and brain magnetic resonance imaging (MRI) within a 90-day interval were eligible for inclusion. The overall analytic cohort comprised 84 participants. LDL-C measurements obtained within 30 days of imaging were available in 69 participants; LDL-based analyses were therefore performed in this complete-case subgroup.

The study protocol was approved by the institutional Non-Interventional Clinical Research Ethics Committee (decision No.: 2025/16/17; date: 29 July 2025). The requirement for informed consent was waived due to the retrospective design and use of anonymized data.

Imaging examinations were performed for routine clinical indications rather than a standardized research protocol, and indications were heterogeneous, which may introduce selection bias.

During the preparation of this manuscript, generative artificial intelligence (ChatGPT, OpenAI, San Francisco, CA, USA; GPT-5.3 version) was used exclusively for language editing and clarity improvement. All scientific content, data interpretation, and final manuscript approval were performed by the authors.

### 2.2. Inclusion and Exclusion Criteria

Adult participants with technically adequate coronary CT and brain MRI examinations were included. Exclusion criteria were poor image quality precluding reliable volumetric segmentation; presence of major intracranial pathology (e.g., brain tumor, large territorial infarction, normal-pressure hydrocephalus); interval between CT and MRI exceeding 90 days; and missing key clinical variables required for multivariable analyses.

### 2.3. Clinical and Laboratory Data

Demographic characteristics and cardiovascular risk factors were retrieved from electronic medical records. Hypertension and diabetes were defined based on documented diagnosis or medication use. Serum lipid measurements, including LDL-C, were obtained from fasting blood samples collected as part of routine care using a direct enzymatic assay (Roche Cobas, Mannheim, Germany). LDL-C was analyzed as a continuous variable (mg/dL). Statin use was recorded as a binary variable and included as a covariate.

### 2.4. Coronary Artery Calcium Scoring

**Figure 1:**
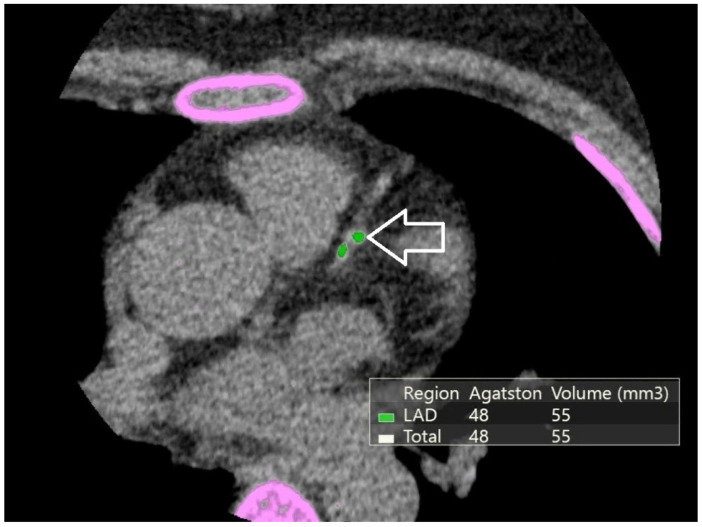
Non-contrast cardiac CT demonstrating coronary artery calcification. Pink overlays indicate software-based segmentation of calcified plaques contributing to Agatston score calculation. The white arrow highlights a representative calcified plaque in the left anterior descending (LAD) artery. The CAC score (Agatston method) serves as a quantitative marker of cumulative atherosclerotic burden.

Non-contrast cardiac computed tomography examinations were performed using a 640-slice computed tomography scanner (Canon Aquilion ONE, Canon Medical Systems, Otawara, Japan) with electrocardiographic synchronization, following a standard calcium-scoring protocol. Coronary artery calcium burden was quantified using the Agatston method, with calcified plaques defined as regions exceeding 130 Hounsfield units. CAC scores were calculated for each participant as a total Agatston score (Figure 1). Due to right-skewed distribution, CAC scores were log-transformed [log (CAC + 1)] for regression analyses. Additionally, CAC was categorized into clinically meaningful groups (0, 1–100, >100 Agatston units (AU)) for categorical analyses.

### 2.5. Brain MRI Acquisition and Volumetric Analysis

Brain MRI examinations were conducted using a 1.5-Tesla scanner (SIGNA Premier, GE Healthcare, Chicago, IL, USA). High-resolution three-dimensional T1-weighted sequences were acquired for volumetric analysis. An experienced radiologist assessed image quality before analysis to exclude scans with significant artifacts.

**Figure 2:**
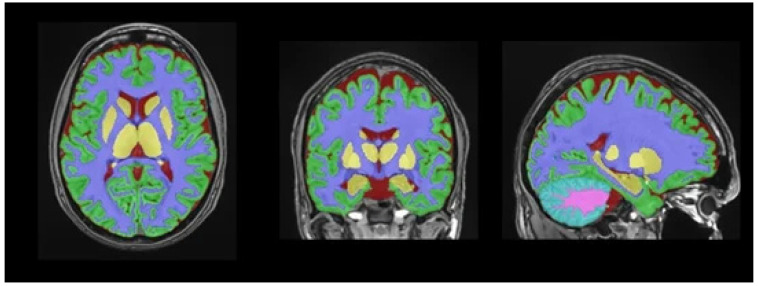
Automated brain segmentation using the volBrain lesionBrain pipeline. T1-weighted image. Segmentation overlay: abnormal white matter (red), normal white matter (white), gray matter (gray), and cerebrospinal fluid (blue). Additional color coding includes pink, representing the cerebellum; green, representing cortical gray matter; purple, representing subcortical white matter regions; and yellow, representing deep gray matter structures (including basal ganglia and thalami).

Automated brain volumetric segmentation and abnormal white matter quantification were performed using the lesionBrain pipeline within the volBrain platform, a previously described automated framework for brain volumetry and white matter lesion segmentation (Figure 2). The visual review of 15 randomly selected cases by an experienced neuroradiologist was used as an internal quality-control step to detect gross segmentation errors and was not intended as a formal validation analysis. Confidence in the imaging outputs is based primarily on previously published methodological studies describing the volBrain/lesionBrain framework [15]. Volumetric measures were analyzed as absolute values and were not normalized to intracranial volume.

The primary outcome was abnormal white matter volume, while secondary outputs included total white matter, total gray matter, subcortical gray matter, CSF, and cerebellar volumes.

### 2.6. Statistical Analysis

Log transformation was applied to improve normality and model fit, and regression coefficients were back-transformed to express results as percentage changes.

Continuous variables were summarized as mean ± SD or median [IQR]. Spearman correlations were performed across all brain regions with Benjamini–Hochberg FDR correction applied separately for each predictor.

Hierarchical multivariable linear regression was performed with log-transformed abnormal white matter volume as the dependent variable, sequentially adding demographic factors, cardiovascular risk factors, LDL-C, statin use, and log-transformed CAC. Both unstandardized and standardized coefficients, along with partial R^2^, were reported. A secondary model assessed gray matter volume.

**Figure 3:**
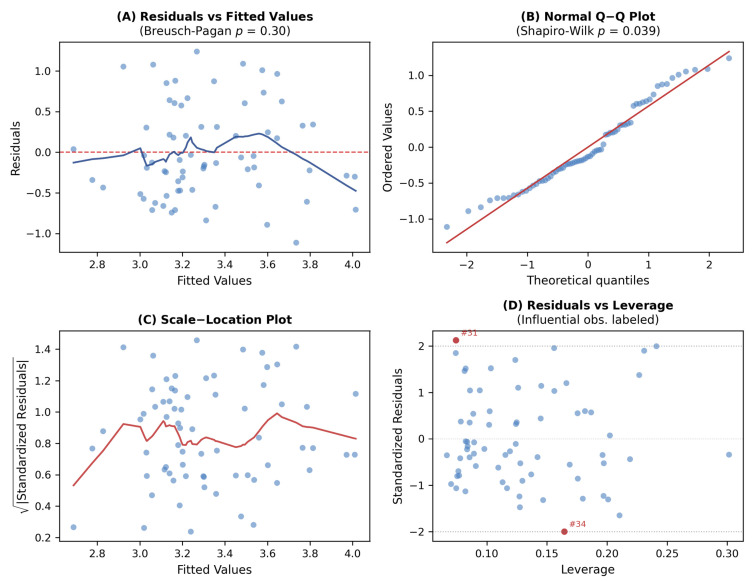
Regression diagnostics for Model 4. (**A**) Residuals vs. fitted values. (**B**) Q-Q plot. (**C**) Scale–location plot. (**D**) Residuals vs. leverage with influential observations labeled.

Post hoc power analysis was performed using Fisher’s z transformation and was used descriptively to contextualize detectable effect sizes and potential type II errors, particularly for CAC. Model assumptions were verified using standard residual diagnostics (Figure 3) (see Supplementary Methods). Influential observations were identified using Cook’s distance (>4/*n*). Bootstrap resampling (2000 iterations) assessed coefficient stability. Model diagnostics are detailed in Supplementary Table S3.

Missing LDL-C values were not imputed. LDL-based analyses used complete-case data (*n* = 69). Participants with and without available LDL-C measurements were compared using the independent-samples *t*-test or Mann–Whitney U test for continuous variables, according to distribution, and the chi-square test or Fisher’s exact test for categorical variables, as appropriate.

Analyses were performed using SPSS 26.0 and Python 3.9. A two-sided *p* < 0.05 was considered statistically significant. This study is reported in accordance with the Strengthening the Reporting of Observational Studies in Epidemiology (STROBE) guidelines [16] (Supplementary Table S5).

## 3. Results

### 3.1. Study Population

After sequential application of the exclusion criteria—poor image quality (*n* = 7), major intracranial pathology (*n* = 18), CT–MRI interval > 90 days (*n* = 29), and missing key clinical variables (*n* = 18)—84 participants comprised the overall analytic cohort. Of the 84 participants, 69 (82.1%) had available LDL-C data. The median CT–MRI interval was 45 days (IQR, 21–78), and the median LDL–MRI interval was 14 days (IQR, 7–28). Participants with missing LDL-C data were younger (median age, 52 vs. 64 years; *p* = 0.009) and had lower CAC scores (median, 1 vs. 4 AU; *p* = 0.028), but did not differ in sex (*p* = 0.79) or abnormal white matter volume (*p* = 0.11), suggesting potential selection bias.

### 3.2. Demographic and Clinical Characteristics

The demographic and clinical characteristics of the study population, stratified by sex, are presented in Table 1. The mean age was 58.0 ± 13.0 years (range, 19–78), and 45 participants (53.6%) were female. Males had significantly higher CAC scores (median 7 [IQR 2–109] vs. 1 [IQR 1–9], *p* = 0.011) and lower prevalence of hypertension (41.0% vs. 71.1%, *p* = 0.011) compared with females. No significant sex differences were observed for age (*p* = 0.980), LDL-C (*p* = 0.094), abnormal white matter volume (*p* = 0.660), diabetes (*p* = 0.628), smoking (*p* = 0.748), or statin use (*p* = 1.000). The mean LDL-C level was 113.1 ± 37.6 mg/dL. The median CAC score was 3 AU (IQR, 1–44); 14.3% had CAC = 0, 65.5% had CAC 1–100, and 20.2% had CAC > 100.

### 3.3. Correlation Analyses

Age showed expected negative correlations with brain parenchymal volumes and a positive correlation with CSF volume (Table 2; complete matrix in Table S1).

**Figure 4:**
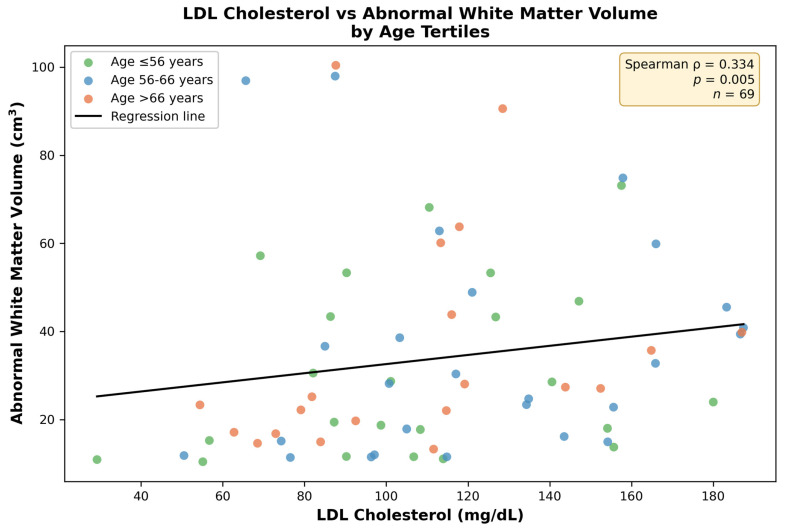
LDL cholesterol versus abnormal white matter volume. Points are colored by age tertile for descriptive visualization only. The age-adjusted association is reported in the multivariable model. Regression line with 95% CI shown. Widening confidence intervals at higher LDL-C levels reflect data sparsity.

LDL-C demonstrated a positive univariable correlation with abnormal white matter volume (ρ = 0.334, *p* = 0.005), but this correlation did not remain statistically significant after FDR correction (pFDR = 0.090 after correction across 18 brain regions) (Figure 4). LDL-C showed no correlation with gray matter volumes (total: ρ = −0.123, *p* = 0.315; subcortical: ρ = −0.011, *p* = 0.925).

CAC showed no significant correlations with any brain region after FDR correction, including abnormal white matter (ρ = 0.086, *p* = 0.439, pFDR = 0.705) and total gray matter (ρ = −0.207, *p* = 0.059, pFDR = 0.215).

### 3.4. Regression Analyses

In hierarchical regression (Table 3), adding LDL-C substantially improved model fit (ΔR^2^ = 0.092). In the final model (R^2^ = 0.205), higher LDL-C was the only variable independently associated with abnormal white matter volume: unstandardized β = 0.006 (95% CI: 0.002–0.010, *p* = 0.007), standardized β = 0.225, and partial R^2^ = 11.7%, corresponding to a 6.2% increase in abnormal white matter volume per 10 mg/dL LDL-C. CAC was not independently associated with abnormal white matter volume in this cohort (β = 0.080, *p* = 0.059). All VIF values were <2.0.

In the secondary analysis with gray matter volume as the outcome (Table 4), neither LDL-C (*p* = 0.256) nor CAC (*p* = 0.275) showed significant associations. Only age (*p* = 0.050) and diabetes (*p* = 0.031) were significant, supporting regional specificity of the LDL–white matter relationship.

### 3.5. Sensitivity Analyses

Seven influential observations (10.1%) were identified using Cook’s distance (>4/*n*) as part of an exploratory sensitivity analysis.

Post hoc power was 80.6% for the LDL–white matter correlation (r = 0.334) but only 12.1% for the CAC correlation (r = 0.086), indicating the study was adequately powered for LDL but underpowered to detect small CAC effects (Table 5).

Seven influential observations (10.1%) were identified (Table 6). After exclusion (*n* = 62), the LDL association strengthened (β = 0.008, *p* = 0.0002), while the CAC association became non-significant (*p* = 0.15). Bootstrap validation (2000 iterations) confirmed LDL coefficient stability: 99.7% positive estimates, 95% CI [0.002, 0.011] excluding zero. In contrast, CAC showed instability (95% CI including zero; Table S4).

## 4. Discussion

In this cross-sectional study, higher LDL-C was independently associated with greater abnormal white matter volume in adjusted analyses. No statistically significant association between CAC and brain volumetric measures was detected in this cohort; however, the CAC analyses were underpowered, and small associations cannot be excluded. Neither LDL-C nor CAC was associated with gray matter volume.

Importantly, the univariable LDL-C–abnormal white matter volume correlation did not remain statistically significant after FDR correction (pFDR = 0.090). The adjusted regression finding should therefore be interpreted cautiously as an association observed within the multivariable model rather than as definitive evidence of a robust univariable signal. Because regression adjustment can alter estimates in either direction, the difference between the univariable and adjusted results should not be overinterpreted and requires confirmation in larger datasets.

The observed association between LDL-C and abnormal white matter volume aligns with emerging evidence implicating dyslipidemia in cerebral small vessel disease [17,18,19]. Our finding aligns with some prior studies linking LDL-C to white matter injury, including those examining LDL-C variability and white matter microstructural integrity, although the broader literature on LDL-C and white matter hyperintensity volume remains heterogeneous [20,21]. Our effect size—a 6.2% increase in white matter lesion volume per 10 mg/dL increase in LDL-C—is consistent with prior population-based studies reporting similar magnitudes of associationThe finding that LDL-C explains approximately 12% of variance in white matter injury (partial R^2^), independent of age and traditional vascular risk factors, supports its potential role as a modifiable contributor to cerebral microvascular pathology.

A key finding of this study is the regional specificity of the LDL–white matter association. LDL-C showed no significant association with gray matter volumes in either univariable or multivariable analyses. This dissociation suggests that lipid-mediated vascular injury may preferentially affect the cerebral microvasculature supplying white matter, particularly the long penetrating arterioles that are highly susceptible to endothelial dysfunction and blood–brain barrier disruption [10,11]. This pattern is consistent with established models of cerebral small vessel disease emphasizing endothelial dysfunction, blood–brain barrier breakdown, and chronic hypoperfusion in white matter [17,18,19]. In contrast, gray matter, characterized by a denser vascular network and greater collateral supply, may exhibit a different vulnerability profile.

The absence of a significant association between CAC and brain volumes warrants cautious interpretation. Post hoc power analysis indicated that the study had only 12% power to detect the observed CAC–white matter correlation (r = 0.086), highlighting a considerable risk of type II error. Large-scale cohort studies, such as MESA (*n* > 6000), have reported modest yet statistically significant associations between CAC and brain outcomes [2,13]. Therefore, the lack of a detectable association in our cohort likely reflects insufficient statistical power rather than a true absence of biological effect. Accordingly, this finding should be interpreted as “not detected” rather than “absent,” underscoring the need for adequately powered studies to more definitively assess CAC–brain relationships.

CAC reflects cumulative and largely stabilized calcified plaque burden, serving as a marker of long-term atherosclerotic exposure and cardiovascular risk [4,5]. However, calcified plaques may not accurately represent ongoing vascular injury relevant to cerebral microcirculation. In contrast, circulating LDL-C is a dynamic and modifiable factor that can induce sustained endothelial dysfunction. This distinction may underlie the differential associations observed in our study. Future longitudinal studies are needed to determine whether either marker is more informative for subsequent white matter change.

From a methodological perspective, prior population-based MRI studies, including those by Bos et al. [12] and Romero et al. [14], have demonstrated associations between systemic atherosclerotic burden and structural brain changes. Xia et al. reported significant associations between coronary artery calcium (CAC) and brain measures in a substantially larger cohort (*n* = 2303) with different demographic characteristics (Dutch population; mean age, 69 years) [2]. In contrast, our younger Turkish cohort (mean age, 58 years) exhibited markedly lower median CAC scores (3 vs. 33 Agatston units in Xia et al.), potentially reflecting a lower overall atherosclerotic burden [2]. This difference may have limited our ability to detect significant CAC–brain relationships. Therefore, these demographic and methodological discrepancies should be considered when interpreting and comparing the findings.

Several limitations warrant emphasis. First, the cross-sectional design precludes causal inference and does not allow assessment of temporal relationships. Second, this was a single-center study at a tertiary-care university hospital; local referral patterns, case-mix, and imaging practices may limit generalizability. Third, LDL-C was measured at a single time point and may not reflect cumulative lipid exposure or duration of dyslipidemia. Fourth, residual confounding is possible because inflammatory biomarkers, indices of blood pressure control, and other measures of vascular burden were not available. Fifth, participants without LDL-C measurements were younger and had lower CAC burden, so the complete-case LDL subgroup may represent a relatively higher-risk subset; this may have influenced the observed LDL-C association and limits generalizability. Given this non-random pattern of missingness and the modest sample size, LDL-C values were not imputed. Sixth, the sample size was modest, limiting power for subgroup analyses and especially for detecting small CAC–brain associations. Seventh, CAC reflects coronary rather than cerebral or carotid atherosclerosis, which may be more directly related to brain structure.

Despite these limitations, the study has several strengths: comprehensive correlation analyses across all brain compartments with appropriate multiple-testing correction; standardized, automated volumetric analysis using validated FLAIR-based white matter hyperintensity segmentation; transparent reporting of negative findings and statistical power limitations; and rigorous sensitivity analyses, including bootstrap validation.

Future research should prioritize longitudinal designs to assess whether LDL-C levels or changes therein predict white matter lesion progression. Studies incorporating repeated lipid measurements, inflammatory biomarkers, and arterial stiffness measures may further elucidate mechanisms. Most importantly, adequately powered studies are needed to characterize CAC–brain relationships definitively.

Beyond LDL-C and CAC, LOX-1-related markers such as circulating soluble LOX-1 may capture oxidized lipid burden, endothelial activation, and vascular inflammation more directly than conventional lipid measures alone. In future heart–brain axis studies, these markers could be evaluated alongside LDL-C, oxidized LDL, CAC, inflammatory biomarkers, and MRI-derived abnormal white matter volume to assess whether they provide complementary information on cardiac–brain vascular injury [22].

## 5. Conclusions

In this cross-sectional study, higher LDL-C was independently associated with greater abnormal white matter volume after adjustment for age, cardiovascular risk factors, statin use, and CAC. No CAC–brain association was detected in this cohort, but the CAC analysis was underpowered, and small effects cannot be excluded. Neither LDL-C nor CAC was associated with gray matter volume. These findings are associative and require confirmation in longitudinal studies evaluating the progression of abnormal white matter volume and the potential impact of lipid-lowering interventions.

## Figures and Tables

**Table 1:** Demographic and clinical characteristics, stratified by sex.

Characteristic	Total (*n* = 84)	Female (*n* = 45)	Male (*n* = 39)	*p*
Age, years, mean ± SD ^a^	58.0 ± 13.0	58.0 ± 11.9	58.0 ± 14.2	0.980
Hypertension, *n* (%) ^b^	48 (57.1)	32 (71.1)	16 (41.0)	0.011 *
Diabetes mellitus, *n* (%) ^b^	27 (32.1)	16 (35.6)	11 (28.2)	0.628
Current smoking, *n* (%) ^c^	11 (13.1)	5 (11.1)	6 (15.4)	0.748
Statin use, *n* (%) ^c^	9 (10.7)	5 (11.1)	4 (10.3)	1.000
LDL-C, mg/dL, mean ± SD ^a,†^	113.1 ± 37.6	120.3 ± 31.8	105.2 ± 42.2	0.094
CAC score, median [IQR] ^d^	3 [1–44]	1 [1–9]	7 [2–109]	0.011 *
CAC = 0, *n* (%)	12 (14.3)	9 (20.0)	3 (7.7)	—
CAC 1–100, *n* (%)	55 (65.5)	29 (64.4)	26 (66.7)	—
CAC > 100, *n* (%)	17 (20.2)	7 (15.6)	10 (25.6)	—
AWM volume, cm^3^, median [IQR] ^d^	27.7 [16.7–50.0]	26.5 [16.2–53.3]	28.5 [18.3–45.1]	0.660

Values are mean ± SD, median [IQR], or *n* (%). * *p* < 0.05. ^†^ LDL-C available subgroup (*n* = 69; 36 female, 33 male). ^a^ Independent-samples *t*-test. ^b^ Chi-square test. ^c^ Fisher’s exact test. ^d^ Mann–Whitney U test. AWM, abnormal white matter; CAC, coronary artery calcium; IQR, interquartile range; LDL-C, low-density lipoprotein cholesterol; SD, standard deviation.

**Table 2:** Spearman correlations between vascular markers and brain volumes.

Brain Region	Age ρ	pFDR	CAC ρ	pFDR	LDL ρ	pFDR
White Matter (total)	−0.255	0.045 *	−0.027	0.908	0.015	0.926
Abnormal White Matter	−0.004	0.972	0.086	0.705	0.334	0.090
Gray Matter (total)	−0.219	0.068	−0.207	0.215	−0.123	0.819
Subcortical GM	−0.250	0.045 *	−0.228	0.215	−0.011	0.926
CSF	0.370	0.009 *	0.282	0.170	0.045	0.918
Cerebellum	−0.316	0.028 *	−0.080	0.705	−0.082	0.819

* pFDR < 0.05. Full correlation matrix available in Supplementary Table S1. CSF, cerebrospinal fluid; GM, gray matter.

**Table 3:** Hierarchical regression for abnormal white matter volume.

Model	R^2^	ΔR^2^	Variable	β (Unstd)	95% CI	β (Std)	*p*
Model 1	0.037	—	Age + Sex				
Model 2	0.064	0.027	+HT, DM, Smoking				
Model 3	0.156	0.092	+LDL-C	0.006	0.001, 0.010	0.225	0.012 *
Model 4	0.205	0.049	LDL-C	0.006	0.002, 0.010	0.225	0.007 **
			Log(CAC + 1)	0.080	−0.003, 0.164	0.177	0.059
			Statin	−0.075	−0.547, 0.397	−0.025	0.752

*n* = 69. * *p* < 0.05, ** *p* < 0.01. Effect: 6.2% AWM increase per 10 mg/dL LDL-C. Partial R^2^: LDL-C = 11.7%, CAC = 5.8%.

**Table 4:** Regression for gray matter volume.

Variable	β (Unstd)	95% CI	β (Std)	*p*
Age	−0.0044	−0.0089, −0.0001	−0.403	0.050 *
LDL-C	−0.0007	−0.0019, 0.0005	−0.119	0.256
Log(CAC + 1)	−0.0129	−0.0364, 0.0105	−0.117	0.275
Diabetes	−0.1111	−0.2117, −0.0105	−0.267	0.031 *

Model R^2^ = 0.206; *n* = 69. * *p* < 0.05. Neither LDL-C nor CAC was associated with gray matter volume.

**Table 5:** Post hoc power analysis.

Analysis	Effect Size	*n*	Power	Interpretation
LDL-AWM correlation	r = 0.334	69	80.6%	Adequately powered
CAC-AWM correlation	r = 0.086	84	12.1%	Underpowered
Minimum detectable r	r = 0.333	69	80%	Threshold met for LDL

Power calculated using Fisher’s z transformation at α = 0.05 (two-tailed).

**Table 6:** Characteristics of influential observations.

Case	Age	Sex	LDL	CAC	AWM	Cook’s D	Reason
1	61	F	154	196	15.0	0.081	Low AWM despite high LDL
2	65	M	88	310	98.0	0.120	High AWM, normal LDL
3	57	F	158	292	74.8	0.062	High LDL and AWM
4	43	F	90	1	53.3	0.140	High AWM, low LDL/CAC
5	75	F	128	6	90.6	0.040	Very high AWM
6	54	M	156	50	13.8	0.088	Low AWM despite high LDL
7	66	M	66	1604	96.9	0.078	Extreme CAC outlier

Cook’s D threshold = 4/*n* = 0.058. AWM, abnormal white matter (cm^3^); CAC, Agatston units; LDL, mg/dL.

## Data Availability

The data that support the findings of this study are available from the corresponding author upon reasonable request. The data are not publicly available due to privacy and ethical restrictions.

## Supplementary Materials

The following supporting information can be downloaded at https://www.mdpi.com/article/10.3390/jcm15093277/s1: Table S1: Complete Spearman correlation matrix; Table S2: Exploratory subgroup analyses; Table S3: Model diagnostics summary; Table S4: Bootstrap validation results; Table S5: STROBE checklist for cross-sectional studies.
