# Supplementary material for: Low-Density Lipoprotein Cholesterol Is Independently Associated with White Matter Injury Beyond Coronary Artery Calcium: Insights into Brain Aging
Source: J Clin Med. 2026 Apr 25;15(9):3277. doi: 10.3390/jcm15093277 (PMC13164002; doi:10.3390/jcm15093277)
Supplement: Supplementary file 1 [file jcm-15-03277-s001.zip › jcm-4239652-supplementary.pdf]

SUPPLEMENTARY MATERIAL

Low-Density Lipoprotein Cholesterol Is Associated with White Matter Injury Independent of Coronary Artery Calcium

Supplementary Methods

S1. Hierarchical Regression Model Structure

Hierarchical multivariable linear regression was performed with log-transformed abnormal white matter volume as the primary dependent variable:

- Model 1: Age + Sex
- Model 2: Model 1 + Hypertension + Diabetes + Smoking
- Model 3: Model 2 + LDL-C
- Model 4: Model 3 + Statin use + log(CAC+1)

Effect sizes were expressed as percentage change in abnormal white matter volume per 10 mg/dL LDL-C increase: $(\exp(\beta \times 10) - 1) \times 100$.

S2. Model Diagnostics

Homoscedasticity was assessed using the Breusch-Pagan test. Autocorrelation was evaluated using the Durbin-Watson statistic (acceptable range: 1.5-2.5). Normality was tested using Shapiro-Wilk test with Q-Q plot visual inspection. Multicollinearity was assessed using variance inflation factors (VIF < 5 acceptable). Influential observations were identified using Cook's distance (threshold: 4/n) and studentized residuals ($|r| > 2$).

S3. Bootstrap Validation

Bootstrap resampling (2,000 iterations) with bias-corrected and accelerated (BCa) percentile confidence intervals was performed. Coefficient stability required: (1) >99% of samples yielding coefficients in hypothesized direction, and (2) 95% BCa CI excluding zero.

S4. Power Analysis

Post-hoc power for Spearman correlations was calculated using Fisher's z transformation: $z = 0.5 \times \ln[(1+r)/(1-r)]$, $SE = 1/\sqrt{(n-3)}$, at $\alpha = 0.05$ (two-tailed).

Supplementary Tables

Table S1. Complete Spearman Correlation Matrix: All Brain Regions

Brain Region	Age ρ	p_FDR	CAC ρ	p_FDR	LDL ρ	p_FDR
Intracranial Volume	-0.089	0.632	-0.045	0.908	0.021	0.926
Brain Parenchyma	-0.259	0.045*	-0.105	0.619	-0.066	0.819
Total White Matter	-0.255	0.045*	-0.027	0.908	0.015	0.926
Total Grey Matter	-0.219	0.068	-0.207	0.215	-0.123	0.819
Cortical GM	-0.207	0.075	-0.206	0.215	-0.143	0.819
Subcortical GM	-0.250	0.045*	-0.228	0.215	-0.011	0.926
Cerebellum Total	-0.316	0.028*	-0.080	0.705	-0.082	0.819
Cerebellar WM	-0.198	0.095	-0.012	0.958	-0.038	0.906
Cerebellar GM	-0.289	0.036*	-0.094	0.665	-0.098	0.819
Brainstem	-0.156	0.202	-0.134	0.448	0.045	0.894
CSF Volume	0.370	0.009*	0.282	0.170	0.045	0.918
Lateral Ventricles	0.342	0.012*	0.245	0.170	0.089	0.819
Abnormal WM	-0.004	0.972	0.086	0.705	0.334	0.090
Left Hemisphere	-0.248	0.045*	-0.118	0.543	-0.078	0.819
Right Hemisphere	-0.267	0.045*	-0.092	0.665	-0.054	0.859
Corpus Callosum	-0.189	0.103	-0.098	0.643	0.067	0.819
Thalamus	-0.278	0.040*	-0.167	0.387	-0.034	0.906
Hippocampus	-0.145	0.226	-0.089	0.665	-0.112	0.819

*p_FDR < 0.05. Age and CAC: n=84; LDL: n=69. CSF, cerebrospinal fluid; GM, grey matter; WM, white matter.

Table S2. Exploratory Subgroup Analyses

Subgroup	n	ρ	p (raw)	p_FDR
Overall sample	69	0.334	0.005	—
Female	37	0.475	0.003	0.027*
Male	32	0.178	0.330	0.495
Age < 60 years	33	0.430	0.012	0.031*
Age ≥ 60 years	36	0.251	0.140	0.252
Hypertension (+)	42	0.379	0.013	0.031*
Hypertension (−)	27	0.264	0.183	0.275
Diabetes (+)	25	0.490	0.013	0.031*
Diabetes (−)	44	0.228	0.137	0.252

*p_FDR < 0.05. Exploratory analyses; study not powered for interaction testing.

Table S3. Model Diagnostics Summary

Test	Statistic	Value	Threshold	Result
Homoscedasticity	Breusch-Pagan	χ^2 = 9.47	p > 0.05	p = 0.30 ✓
Autocorrelation	Durbin-Watson	2.21	1.5-2.5	✓
Normality	Shapiro-Wilk	W = 0.963	p > 0.05	p = 0.039 (marginal)
VIF (Age)	—	1.18	< 5.0	✓
VIF (LDL)	—	1.35	< 5.0	✓
VIF (CAC)	—	1.88	< 5.0	✓
Influential cases	Cook's D	7 cases	> 4/n	Table 6

VIF, variance inflation factor. All VIF values ranged 1.18-1.88.

Table S4. Bootstrap Validation Results (2,000 Iterations)

Variable	Original β	Bootstrap Mean	95% BCa CI	% Positive	Stable
LDL-C	0.006	0.006	[0.002, 0.011]	99.7%	Yes
Log(CAC+1)	0.080	0.078	[-0.015, 0.170]	95.2%	No
Statin use	-0.075	-0.093	[-0.630, 0.443]	36.8%	No
Age	0.002	0.002	[-0.016, 0.019]	58.4%	No
Sex (male)	0.141	0.136	[-0.214, 0.486]	78.2%	No
Hypertension	0.089	0.094	[-0.291, 0.476]	68.5%	No
Diabetes	-0.196	-0.189	[-0.558, 0.179]	15.3%	No
Smoking	0.191	0.180	[-0.321, 0.681]	76.1%	No

BCa, bias-corrected and accelerated. Stability required >99% positive AND 95% CI excluding zero.

Table S5. STROBE Statement—Checklist of Items for Cross-Sectional Studies

Item No	Item	Recommendation	Reported on
**Title and Abstract**			
1	**Title and Abstract**	(a) Indicate study design in title/abstract. (b) Provide informative summary.	Title; Abstract
**Introduction**			
2	**Background/rationale**	Explain the scientific background and rationale.	Introduction, paragraphs 1–5
3	**Objectives**	State specific objectives.	Introduction, final paragraph
**Methods**			
4	**Study design**	Present key elements of study design early.	Methods, Section 2.1
5	**Setting**	Describe setting, locations, and relevant dates.	Methods, Section 2.1 (single-center; Jan 2020–Dec 2023)
6	**Participants**	Give eligibility criteria and methods of selection.	Methods, Section 2.2
7	**Variables**	Define all outcomes, exposures, predictors, confounders.	Methods, Sections 2.3–2.5
8	**Data sources**	Give sources of data and details of assessment.	Methods, Sections 2.3, 2.4, 2.5
9	**Bias**	Describe efforts to address potential sources of bias.	Methods, Section 2.6; Discussion, Limitations
10	**Study size**	Explain how the study size was arrived at.	Methods, Section 2.1 (retrospective inclusion of all eligible patients during study period); Results, Section 3.5 (post-hoc power analysis, Table 5)
11	**Quantitative variables**	Explain how quantitative variables were handled.	Methods, Section 2.6
12	**Statistical methods**	(a) All methods. (b) Subgroups. (c) Missing data. (d)	Methods, Section 2.6; Supplementary S1–S4

		Sampling. (e) Sensitivity.	
**Results**			
13	**Participants**	(a) Numbers at each stage. (b) Reasons for non-participation. (c) Flow diagram.	Results, Section 3.1
14	**Descriptive data**	(a) Participant characteristics. (b) Missing data counts.	Results, Section 3.2; Table 1 (sex-stratified; LDL missing n = 15)
15	**Outcome data**	Report outcome summary measures.	Results, Section 3.2; Table 1
16	**Main results**	(a) Adjusted/unadjusted estimates with 95% CI. (b) Categories. (c) Clinical measures.	Results, Sections 3.3–3.4; Tables 2–4
17	**Other analyses**	Report subgroup, interaction, sensitivity analyses.	Results, Section 3.5; Tables 5–6; Tables S2, S4
**Discussion**			
18	**Key results**	Summarize key results.	Discussion, paragraph 1
19	**Limitations**	Discuss limitations and potential bias.	Discussion, Strengths and Limitations
20	**Interpretation**	Give cautious overall interpretation.	Discussion, paragraphs 2–5
21	**Generalisability**	Discuss external validity.	Discussion, Limitations
**Other Information**			
22	**Funding**	Give source of funding and role of funders.	Funding statement

Adapted from the STROBE guidelines for cross-sectional studies (von Elm et al., Lancet 2007;370:1453–1457).
